# Women experience lower postprandial oxidative stress compared to men

**DOI:** 10.1186/2193-1801-2-553

**Published:** 2013-10-22

**Authors:** Richard J Bloomer, Sang-Rok Lee

**Affiliations:** Cardiorespiratory/Metabolic Laboratory, Department of Health and Sport Sciences, University of Memphis, Memphis, TN USA; Department of Health and Sport Sciences, The University of Memphis, 106 Roane Field House, Memphis, TN 38152 USA

**Keywords:** Triglycerides, Lipids, Free radicals, Nutrition, Sex

## Abstract

**Background:**

Women have enhanced triglyceride (TAG) removal from the circulation following consumption of high-fat loads, potentially leading to decreased reactive oxygen and nitrogen species (RONS) generation. This may have implications related to long-term health outcomes. We examined the oxidative stress response to high-fat feeding between men and women to determine if women are less prone to postprandial oxidative stress as compared to men.

**Methods:**

A total of 49 women (mean age: 31 ± 12 yrs) and 49 men (mean age: 27 ± 9 yrs) consumed a high-fat meal in the morning hours following a 10–12 hour overnight fast. Blood samples were collected before and at 2 and 4 hours after the meal. Samples were analyzed for TAG, various markers of oxidative stress (malondialdehyde [MDA], hydrogen peroxide [H_2_O_2_], Advanced Oxidation Protein Products [AOPP], nitrate/nitrite [NOx]), and Trolox-Equivalent Antioxidant Capacity (TEAC). Area under the curve (AUC) was calculated for each variable. Effect size calculations were performed using Cohen’s *d*. Data from the total sample of 98 subjects were collected as a part of six previously conducted studies in our lab focused on postprandial oxidative stress, between 2007 and 2012.

**Results:**

AUC was higher for men compared to women for TAG (249.0 ± 21.5 vs. 145.0 ± 9.8 mg·dL^-1^·4 hr^-1^; p < 0.0001; effect size = 0.89), MDA (2.7 ± 0.2 vs. 2.2 ± 0.1 μmol·L^-1^·4 hr^-1^; p = 0.009; effect size = 0.47), H_2_O_2_ (29.9 ± 2.4 vs. 22.5 ± 1.6 μmol·L^-1^·4 hr^-1^; p = 0.001; effect size = 0.55), AOPP (92.8 ± 6.9 vs. 56.4 ± 3.7 μmol·L^-1^·4 hr^-1^; p < 0.0001; effect size = 1.38), and TEAC (1.7 ± 0.1 vs. 1.3 ± 0.0 mmol·L^-1^·4 hr^-1^; p = 0.002; effect size = 0.91). No significant difference was noted for NOx (42.2 ± 4.6 vs. 38.3 ± 3.5 μmol·L^-1^·4 hr^-1^ for men and women, respectively; p = 0.09; effect size = 0.17).

**Conclusion:**

In the context of the current design, women experienced lower postprandial oxidative stress compared to men. Future work is needed to determine the potential health implications of lower postprandial oxidative stress in women.

## Introduction

Oxidative stress can occur when the production of reactive oxygen and nitrogen species (RONS) overwhelms antioxidant defenses (Bloomer 2008). This scenario is well-documented in human studies in which participants ingest a high-fat load (Bell et al. 2010; Bloomer and Fisher-Wellman 2009a; Bloomer and Fisher-Wellman. 2009b; Bloomer et al. 2010b; Melton et al. 2009; Yang et al. 2008; Zhang et al. 2005). The increased oxidative stress is evidenced by the rise in oxidized molecules during the several hour period (e.g., 2–6 hours) immediately following consumption of the meal (Sies et al. 2005)—including but not limited to oxidized glutathione, protein carbonyls, advanced oxidation protein products (AOPP), isoprostanes, malondialdehyde (MDA), hydrogen peroxide (H_2_O_2_), and nitrate/nitrite (NOx). A decrease in selected markers of antioxidant status has also been observed (McCarthy et al. 2013).

The oxidation of important cellular components has been implicated in a variety of human diseases (Dalle-Donne et al. 2006; Halliwell and Cross. 1994; Valko et al. 2007), in addition to the aging process (Pamplona and Barja 2011; Sena and Chandel 2012; Yin et al. 2012). While the production of RONS occurs as part of normal cellular metabolism (Fialkow et al. 2007; Stanczyk et al. 2005), in particular through the processing of NADH and FADH_2_ in the electron transport chain, excess RONS production can be problematic; in particular as related to cardiovascular (Victor et al. 2009) and metabolic (Kojda and Harrison 1999; Wei et al. 2009) disease.

One activity that appears ubiquitous in terms of increasing oxidative stress is the ingestion of high-fat meals (McCarthy et al. 2013). Considering that many individuals consume such meals on a regular basis, they may exist in a prolonged postprandial state and place themselves at increase risk for disease (Ceriello et al. 2002; Zilversmit 1979).

Two main factors appear to dictate the degree of oxidative stress experienced following high-fat meal ingestion. First, the increase in circulating triglycerides (TAG) triggers an increase in RONS formation (Bae et al. 2001; Bloomer et al. 2010a)—with elevated TAG strongly correlated to the rise in oxidized macromolecules (Bloomer et al. 2010b; Fisher-Wellman and Bloomer 2010; McCarthy et al. 2013). Second, impaired antioxidant defenses lead to further elevation in oxidized macromolecules (Bonnefont-Rousselot et al. 2000), as insufficient protection is available in the presence of increased RONS.

Considering the above, it is believed that women are less susceptible to postprandial oxidative stress than men due to exhibiting enhanced TAG removal from the circulation following feeding, as well as having relatively higher concentrations of estrogen (which provide antioxidant protection). Indeed, women have been shown to exhibit improved TAG clearance following high-fat feeding as compared to men (Couillard et al. 1999), with diminishing benefit following menopause (van Beek et al. 1999)—strongly suggesting a role of estrogen in mediating this effect. Estrogen also provides antioxidant protection (Mendelsohn and Karas 1999), possibly resulting in lower oxidative stress following an acute elevation in RONS. Mechanistically, estrogen binds estrogen receptors and stimulates mitogen activated protein kinase and NF-kB signaling pathways to induce an up-regulation in endogenous antioxidants (Vina et al. 2006). In support of this, it has been reported that mitochondria from female rats exhibit higher antioxidant enzyme gene expression compared to male rats, in addition to higher mitochondrial glutathione (Borras et al. 2003).

For the above reasons, it is believed that women are less susceptible to postprandial oxidative stress as compared to men. This paper presents data relative to the oxidative stress response between men and women during the four-hour period following the ingestion of a high-fat meal.

## Methods

### Subjects

A total of 49 men and 49 women were included in this study. Data from the total sample of 98 subjects were collected as a part of six previously conducted studies in our lab focused on postprandial oxidative stress, between 2007 and 2012. Subjects completed a health history and physical activity questionnaire prior to being enrolled. No subject was a current smoker or had a history of cardiovascular or metabolic disease. Subjects were healthy and young (mean age <30 years old), of usual body weight (mean of 82 ± 8 kg for men; mean or 75 ± 21 kg for women), with most subjects claiming (based on self report) to participate in a program of structured exercise (e.g., 2–4 days per week of aerobic exercise and/or resistance exercise). All but three subjects (all men) presented with fasting serum TAG values <150 mg·dL^-1^. All experimental procedures were approved by The University Human Subjects Institutional Review Board. Subjects provided written informed consent prior to participating.

### Test meals

Testing of all subjects was performed in the morning hours following an overnight fast (10–12 hours). Subjects consumed a high-fat milkshake, consisting of approximately 1.0 gram of fat (approximately 65% saturated fat), 1.0 gram of carbohydrate, and 0.25 grams of protein per kilogram of body weight. The dosage of fat provided was similar to that used in other studies of postprandial lipemia and supported by recent recommendations for oral fat tolerance testing (Kolovou et al. 2011). The milkshake was made using whole milk, Breyers® “all natural” vanilla ice cream, and heavy whipping cream. Subjects were allowed 15 minutes for complete ingestion. Subjects remained in the laboratory (or in close proximity) during the four-hour postprandial data collection period and rested (i.e., watched movies, read, listened to music, worked on a computer, etc.). No additional meals or calorie containing beverages were allowed. Water was allowed *ad libitum*.

### Blood collection and analysis

For all subjects at each time of collection (pre meal, 2 and 4 hours post meal) approximately 10 mL of blood was taken from a forearm vein via needle and collection tube. The four-hour sampling time is supported by recent recommendations for the assessment of postprandial lipemia (Mihas et al. 2011). Blood collected in tubes containing EDTA was centrifuged immediately at 4°C and plasma was stored in multiple aliquots at -70°C until analyzed. Blood collected in tubes with no additive was allowed to clot at room temperature for 30 minutes and then centrifuged at 4°C. The serum was stored in multiple aliquots at -70°C until analyzed.

Triglyceride (TAG) was analyzed in serum following enzymatic procedures as described by the reagent provider (Thermo Electron Clinical Chemistry). Malondiadehyde (MDA) was analyzed in plasma using a commercially available colorimetric assay (Northwest Life Science Specialties, Vancouver, WA), using similar methods as previously described (Jentzsch et al. 1996). It should be noted that samples for MDA analysis were only available for 48 men. Hydrogen peroxide (H_2_O_2_) was analyzed in plasma using the Amplex Red reagent method as described by the manufacturer (Molecular Probes, Invitrogen Detection Technologies, Eugene, OR). It should be noted that samples for H_2_O_2_ analysis were only available for 43 men and 48 women. Advanced Oxidation Protein Products (AOPP) were analyzed in plasma using methods previously described (Witko-Sarsat et al. 1996). It should be noted that samples for AOPP analysis were only available for 24 men and 19 women. Nitrate/nitrite (NOx) was analyzed using a commercially available colorimetric assay kit (Cayman Chemical, Ann Arbor, MI) according to the procedures provided by the manufacturer. It should be noted that samples for NOx analysis were only available for 30 men and 33 women. Antioxidant capacity was analyzed using the Trolox-Equivalent Antioxidant Capacity (TEAC) assay using procedures outlined by Sigma Chemical (St. Louis, MO). It should be noted that samples for TEAC analysis were only available for 47 men and 48 women. As indicated previously, biomarker values from subjects were pooled from a series of individual studies of postprandial oxidative stress as performed in our lab. Therefore, actual values for each biomarker were not available for every subject.

### Dietary records and physical activity

All subjects were instructed to maintain their usual diet and physical activity habits leading up to their test day, with one exception: they were asked to refrain from strenuous activity during the 24 hours prior to testing. This was important in an attempt to control for any potential acute effects of physical activity on postprandial lipemia and oxidative stress (Mc Clean et al. 2007).

### Statistical analysis

Area under the curve (AUC) was calculated for each variable using the trapezoidal method (AUC_G_) as described in detail by Pruessner and colleagues (Pruessner et al. 2003). Variables were then analyzed using a one way analysis of variance (ANOVA). Effect size calculations were performed using Cohen’s *d*. Data were also analyzed using a 2 (sex) by 3 (time) ANOVA, with Tukey post hoc testing as needed. Outcome data are presented as mean ± standard error of the mean. Analyses were performed using JMP statistical software (version 4.0.3, SAS Institute, Cary, NC). Statistical significance was set at p ≤ 0.05.

## Results

### Oxidative stress biomarker data: area under the curve

The AUC was higher for men compared to women for TAG (249.0 ± 21.5 vs. 145.0 ± 9.8 mg·dL^-1^·4 hr^-1^; F = 20.6; p < 0.0001; effect size = 0.89), MDA (2.7 ± 0.2 vs. 2.2 ± 0.1 μmol·L^-1^·4 hr^-1^; F = 7.6; p = 0.009; effect size = 0.47), H_2_O_2_ (29.9 ± 2.4 vs. 22.5 ± 1.6 μmol·L^-1^·4 hr^-1^; F = 12.2; p = 0.001; effect size = 0.55), AOPP (92.8 ± 6.9 vs. 56.4 ± 3.7 μmol·L^-1^·4 hr^-1^; F = 20.0; p < 0.0001; effect size = 1.38), and TEAC (1.7 ± 0.1 vs. 1.3 ± 0.0 mmol·L^-1^·4 hr^-1^; F = 11.7; p = 0.002; effect size = 0.91). No significant difference was noted for NOx (42.2 ± 4.6 vs. 38.3 ± 3.5 μmol·L^-1^·4 hr^-1^ for men and women, respectively; F = 2.9; p = 0.09; effect size = 0.17). Effect size calculations were noted to be large for TAG, AOPP, and TEAC, while being medium for MDA and H_2_O_2_. The effect size calculation for NOx was noted to be small.

### Oxidative stress biomarker data: sex by time ANOVA

**Figure 1:**
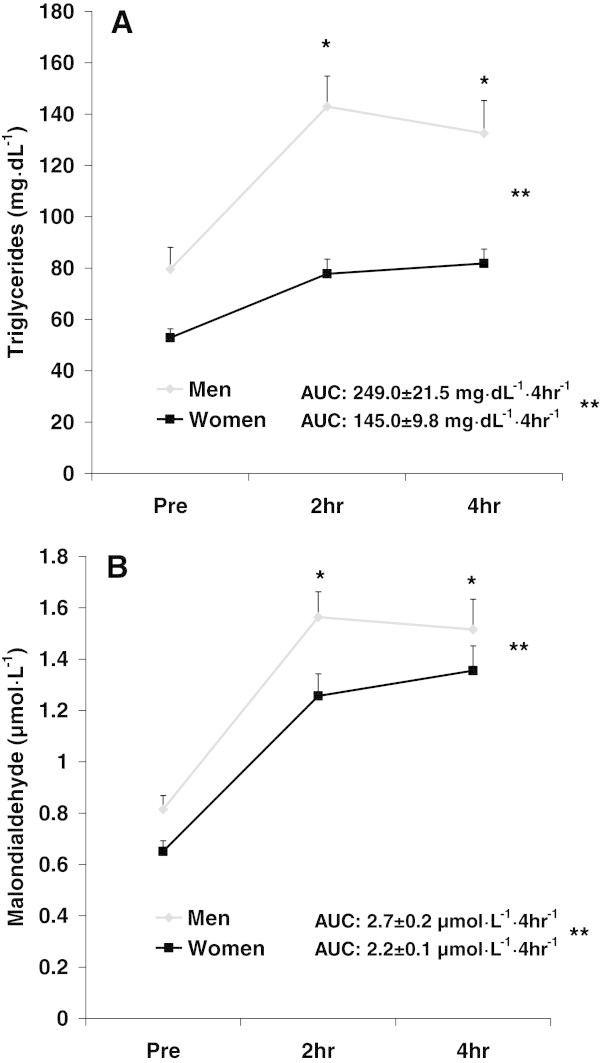
**Triglycerides (A) and malondialdehyde (B) before and following intake of a high-fat meal in men and women.** Values are mean ± SEM. Triglycerides: **Significant difference noted for AUC (p < 0.0001). **Significant effect for sex (p < 0.0001). * Significant effect for time (p = 0.0005); 2 hr and 4 hr greater than Pre. Malondialdehyde: **Significant difference noted for AUC (p = 0.009). **Significant effect for sex (p = 0.0005). * Significant effect for time (p < 0.0001); 2 hr and 4 hr greater than Pre. N = 48 men; N = 49 women.

For TAG, a sex effect was noted (F = 54.1; p < 0.0001), with values higher for men compared to women. A time effect was also noted (F = 8.1; p = 0.0005), with values higher at 2 hr and 4 hr compared to Pre (baseline—prior to meal ingestion). No interaction effect was noted (F = 1.7; p = 0.19). Data for TAG are presented in Figure 1A.

For MDA, a sex effect was noted (F = 12.7; p = 0.0005), with values higher for men compared to women. A time effect was also noted (F = 20.3; p < 0.0001), with values higher at 2 hr and 4 hr compared to Pre. No interaction effect was noted (F = 1.0; p = 0.37). Data for MDA are presented in Figure 1B.

**Figure 2:**
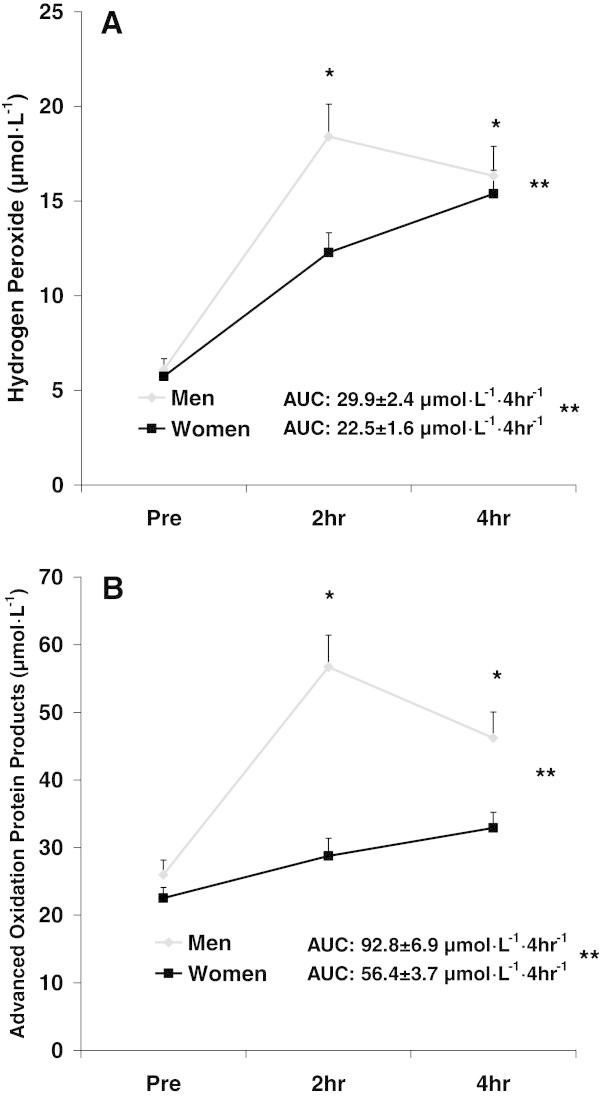
**Hydrogen peroxide (A) and advanced oxidation protein products (B) before and following intake of a high-fat meal in men and women.** Values are mean ± SEM. Hydrogen Peroxide: **Significant difference noted for AUC (p = 0.001). **Significant effect for sex (p < 0.0001). *Significant effect for time (p < 0.0001); 2 hr and 4 hr greater than Pre. N = 43 men; N = 48 women. Advanced Oxidation Protein Products: **Significant difference noted for AUC (p < 0.0001). **Significant effect for sex (p < 0.0001). *Significant effect for time (p < 0.0001); 2 hr and 4 hr greater than Pre. N = 24 men; N = 19 women.

For H_2_O_2_, a sex effect was noted (F = 20.2; p < 0.0001), with values higher for men compared to women. A time effect was also noted (F = 20.3; p < 0.0001), with values higher at 2 hr and 4 hr compared to Pre. An interaction effect was noted (F = 5.9; p = 0.004), with 2 hr lower for women compared to men (p < 0.05). Data for H_2_O_2_ are presented in Figure 2A.

For AOPP, a sex effect was noted (F = 34.7; p < 0.0001), with values higher for men compared to women. A time effect was also noted (F = 17.7; p < 0.0001), with values higher at 2 hr and 4 hr compared to Pre. An interaction effect was noted (F = 6.3; p = 0.003), with 2 hr and 4 hr lower for women compared to men (p < 0.05). Data for AOPP are presented in Figure 2B.

**Figure 3:**
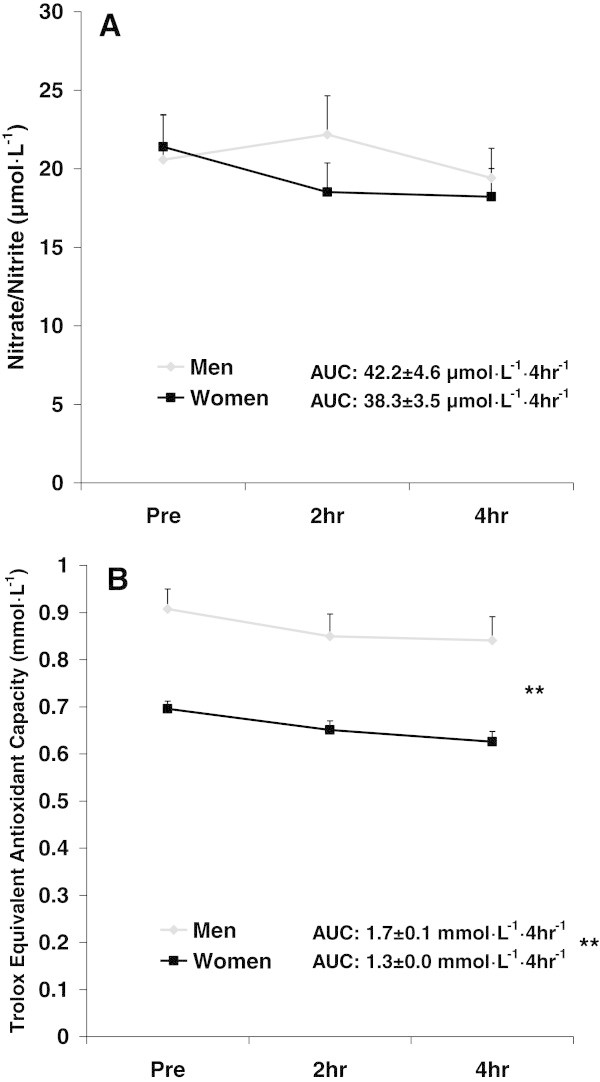
**Plasma nitrate/nitrite (A) and Trolox Equivalent Antioxidant Capacity (B) before and following intake of a high-fat meal in men and women.** Values are mean ± SEM. Nitrate/Nitrite: No significant differences noted (p > 0.05). N = 30 men; N = 33 women. Trolox Equivalent Antioxidant Capacity: **Significant difference noted for AUC (p = 0.002). **Significant effect for sex (p < 0.0001). N = 47 men; N = 48 women.

For NOx, no sex effect (F = 3.7; p = 0.06), time effect (F = 0.1; p = 0.89), or interaction effect (F = 2.1; p = 0.12) was noted. Data for NOx are presented in Figure 3A.

For TEAC, a sex effect was noted (F = 36.7; p < 0.0001), with values higher for men compared to women. No time effect (F = 0.5; p = 0.59) or interaction effect (F = 0.0; p = 0.99) was noted. Data for TEAC are presented in Figure 3B.

## Discussion

Data from the present investigation indicate that women experience a lower oxidative stress response compared to men, following ingestion of a high-fat meal. This is evidenced by a significantly lower AUC for MDA, H_2_O_2_, and AOPP. These data may have health and longevity implications specific to oxidative stress-related disease. Future study is needed to investigate this possibility.

As noted in our prior work (Bloomer et al. 2010b; Fisher-Wellman and Bloomer. 2010; McCarthy et al. 2013), the rise in circulating TAG following meal ingestion appears to dictate the oxidative stress response. As shown in Figure 1A, women experienced a blunted response to feeding with regards to TAG—while also having a lower pre-meal TAG value as compared to men (although not statistically different). This lesser increase in TAG was duplicated for MDA, H_2_O_2_, and AOPP—all of which were significantly lower in women as compared with men. Interestingly, NOx only rose slightly in men and actually decreased slightly in women following feeding, while TEAC decreased slightly in both men and women.

From a mechanistic perspective, although we are uncertain as to exactly why women experienced a much blunted oxidative stress response as compared to men, we propose the following for consideration. First, a correlation exists between the systemic TAG response to feeding and the increase in RONS production and subsequent oxidative stress biomarkers. In the present study we noted a lower TAG response to feeding in women compared to men. These findings agree with those of Kolovou et al. (Kolovou et al. 2006) who observed greater postprandial TAG with delayed TAG clearance in men compared to women. It is certainly conceivable that a blunted postprandial TAG response to feeding ameliorates postprandial oxidative stress in women.

Another consideration is the ability of estrogen to provide antioxidant protection in women. Sugioka and colleagues (Sugioka et al. 1987) reported that estrogen administration reduced lipid peroxidation, possibly by providing hydrogen atoms to lipids within cell membranes, thus accelerating termination of radical reactions. Moreover, Shwaery et al. (Shwaery et al. 1997) found that 17β-estradiol administration antagonized oxidative modification of low density lipoprotein. The higher mean concentrations of estrogen in women may be partly responsible for the lower postprandial oxidative stress response in women compared to men.

Related to the above, it should be noted that we made no attempt to control for menstrual cycle phase in the present study. It is logical to hypothesize that since estrogen fluctuates considerably across the cycle, our goal should have been to test women when estrogen levels are highest (i.e., pre-ovulation). However, we have previously conducted a study in which we tested women at different times during the menstrual cycle to determine the direct influence of estrogen on postprandial lipemia and oxidative stress, noting no differences in outcome measures regardless of when women were tested (Bell et al. 2010). Rather than estrogen concentration at the actual time of testing, what may be of greatest importance is the chronically higher mean levels of estrogen across the entire menstrual cycle that may prevent oxidative damage and may serve as a signal for the up-regulation in endogenous antioxidant defense mechanisms, which then remains present throughout the entire cycle. Of course, well-controlled experiments are needed to confirm this hypothesis. Our failure to measure estrogen concentrations in our subjects may be considered a limitation of this work.

A consideration in interpreting our findings is the degree of adiposity of our subjects, as this has been noted in prior studies to influence outcome measures related to postprandial oxidative stress (Bloomer and Fisher-Wellman 2009a). Unfortunately, we did not have detailed information related to adiposity based on DXA or CT scans. Future studies may aim to include these measures when comparing the postprandial oxidative stress response between men and women. That said, it should be noted that in our prior work with men and women, whom were relatively lean, we also noted a blunted postprandial oxidative stress response in women as compared to men (Bloomer et al. 2009).

We aimed to include a wide variety of oxidative stress biomarkers in the present analysis—those that are used widely throughout the feeding and oxidative stress literature. That said, our presentation may have been strengthened by the inclusion of further markers such as glutathione and isoprostanes, as well as enzymatic antioxidants. Additionally, although we measured biomarkers in blood samples, we are uncertain of the potential differences observed in other tissue such as skeletal muscle and liver. Future study using animal models may allow for an assessment in other tissues. Finally, we ceased our measurement time at four hours post meal ingestion. Although our timing is well-supported (Mihas et al. 2011), values for certain variables were shown to be highest at this time. Therefore, it is possible that values may have continued to increase beyond four hours, potentially altering the differences observed between men and women. Future study may seek to extend the time of collection following high-fat meal ingestion.

## Conclusion

We report that women are less susceptible to postprandial oxidative stress compared to men, following ingestion of a high-fat milkshake. It is possible that lower oxidative stress may be one mechanistic link to lower disease risk and increased longevity in women compared to men (Vina et al. 2006). Future work is needed to confirm this hypothesis.
